# Provision of a daily high protein and high energy meal: Effects on the physical and psychological wellbeing of community-dwelling, malnourished older adults; a randomised crossover trial

**DOI:** 10.1016/j.jnha.2024.100429

**Published:** 2024-12-10

**Authors:** Lauren Struszczak, Mary Hickson, Irene McClelland, Brad Metcalf, Manuela Barreto, Luciana Torquati, Jon Fulford, Rachael Allen, Claire Hulme, Mary F. O’Leary, Joanna L. Bowtell

**Affiliations:** a University of Exeter, Faculty of Health & Life Sciences, St Lukes Campus, Heavitree Road, Exeter EX1 2LU, United Kingdom; b University of Plymouth, School of Health Professions, InterCity Place, North Road East, Plymouth, PL4 6AB, United Kingdom; c Department of Nutrition and Dietetics, Hengrave House, Torbay Hospital, Torquay TQ2 7AA, United Kingdom

**Keywords:** High-protein, Age care, Malnutrition, Sarcopenia

## Abstract

**Objectives:**

To determine whether daily provision of a high protein, high energy meal for 12-weeks to under-nourished older adults living independently in the community can improve physical, physiological, and psychological outcomes.

**Design:**

A randomised crossover trial.

**Setting:**

Participant homes within a 15-mile radius to meal supplier; Dartmoor Community Kitchen Hub.

**Participants:**

Fifty-six community dwelling older adults (82 ± 7 years, 70% female) were randomised (stratified for baseline mini nutritional assessment (MNA) score and cohabiting or living alone) to receive 12-weeks of meal provision followed by 12-weeks no intervention (meals first group, *n* = 28), or, 12-weeks without intervention followed by 12-weeks of meal provision intervention (meals second group, *n* = 28).

**Intervention:**

A daily high protein and high energy home-delivered meal for 12-weeks. Each meal contained >50% daily protein requirements (0.6 g kg^−1^ of the recommended 1.2 g kg^−1^.day^−1^, ∼42 g protein per meal) and >40% daily energy requirements (∼715 kcal).

**Measurements:**

Physical, physiological and psychological health (including MNA score, body composition, hand grip strength, self-esteem, and depression) were evaluated in participants’ homes before and after each 12-week period (baseline, 12-weeks, and 24-weeks). The effect of meal provision was assessed by *t*-test then effects were combined using meta-analysis. Retention of any meal provision effect after cessation of meal delivery was quantified as change from the end of the meal intervention versus 12-weeks follow-up via paired *t*-test.

**Results:**

The meal intervention significantly increased MNA score with a medium effect size (MNA: pooled Cohen’s D = 0.74, *p* < 0.001). Energy and protein intake increased significantly during the control period where participants were asked to maintain their habitual diet in the meals second group (energy intake: increase = 252 kcal [95% CI 36–487 kcal], t(22) = 2.408, *p* = 0.025, protein intake: increase = 0.20 g kg^−1^ [95% CI 0.04–0.357 g kg^−1^], t(22) = 2.629, *p* = 0.015), which confounded the principle of a randomised crossover design analysis. When the control effect in those in the meals second group was removed from the analysis, the effect of the meal provision was much greater (meal provision significantly improved energy and protein intakes (311 kcal *D* = 0.52 (95% CI 0.22 to 0.82), *p* < 0.001; 0.24 g kg^−1^*D* = 0.52 (0.19–0.81), *p* < 0.001, respectively), MNA score (2.6 points *D* = 1.14 (0.78–1.50), *p* < 0.001), and handgrip strength (1.5 kg *D* = 0.36 (0.06–0.66), *p* = 0.02), but did not change levels of depression or self-esteem). Twelve weeks after meal removal, the following % of the meal effect was retained: 68% for MNA score, 27% for negative mood score, 15% for daily energy intake, 6% for daily protein intake and 0% for handgrip strength.

**Conclusion:**

Provision of high protein, high energy meals to community dwelling older adults for 12-weeks improved nutritional status and handgrip strength, indicative of reduced frailty risk. Benefits were not retained upon withdrawal of the intervention, suggesting a need for sustained interventions in this cohort to meet nutritional needs. Home-delivered meals offer a popular, and scalable intervention for community dwelling older adults to prevent malnutrition, promote health and sustain high quality independent living thus reducing the burden of ageing and frailty on health and social care systems.

## Highlights

• 12-weeks meal provision increased daily energy and protein intake in older adults.
• 12-weeks meal provision improved nutritional status in older adults.
• 12-weeks meal provision improved hand grip strength in older adults.
• Benefits are lost within 12 weeks of removal of meal provision.

## Graphical abstract

Created in BioRender. Struszczak, L. (2024) BioRender.com/u65f116.

**Figure:**
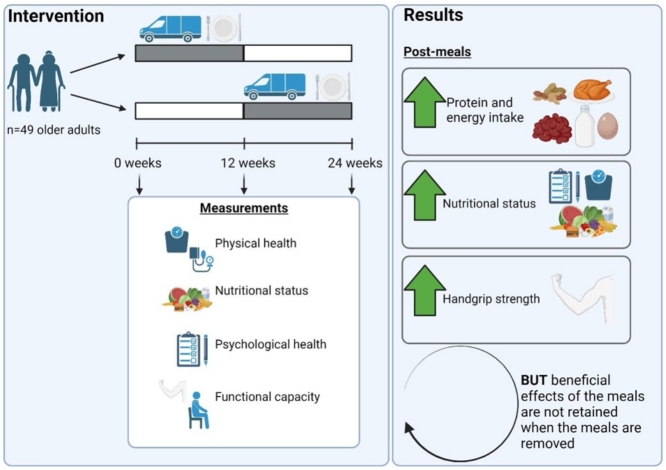

## 1 Introduction

Improvements in living standards, medical advances and public health initiatives, are driving an ageing society. The percentage of the global population >65 years is expected to rise from 10% in 2022 to 16% in 2050 [1]. Yet, life expectancy is not matched by *healthy* life expectancy. Older people have a higher burden of multiple morbidities that affect overall wellbeing and mortality [2]. Food insecurity is rising [3]. In fact, ∼20% of people >65 years old in the UK live in poverty, with finances and social connections worsening for current over 50 s versus previous generations [4].

Poor diet quality and insufficient nutrient intake negatively impact quality of life and mortality risk for community-dwelling older adults [5,6]. Globally, ∼20% of ≥50 s are considered undernourished, frail, or sarcopenic (a condition characterised by the age-related loss of lean mass) [7]. Notably, in the UK, ∼93% of older adults who are at risk of malnutrition, or are already malnourished, are community-dwelling [8]. Malnutrition is associated with higher rates of frailty, surgical complications and longer inpatient admissions [9], further decreasing quality of life and life expectancy [10]. Consequently this leads to a loss of independence and increased use of health and social care resources [11]. Causes of malnutrition are complex but include loss of appetite and taste, difficulties in chewing and swallowing, side effects of medication, reduced capacity to access and prepare food, financial hardship, social isolation, deterioration in psychological wellbeing and the presence of morbidities that alter protein-energy balance [12]. Further, social factors such as isolation and loneliness can exacerbate the risk of malnutrition and associated decreases in health status and quality of life [13].

In contrast, consumption of a high-quality diet meeting and/or exceeding dietary recommendations is associated with better health related outcomes and reduced healthcare resource use [14]. Epidemiological evidence indicates that protein intake is negatively associated with both functional capacity and sarcopenia [15], and therefore decreases risk of cardiovascular [16] and metabolic diseases [17] and incidence of falls and fractures [18]. The UK daily recommended dietary protein intakes for >75-year-olds are, for males/females, 53.3/46.5 g [[19], [20], [21]] which is not dissimilar to recommended dietary intake guidelines for 19–64-year-olds 55.5/45 g. This is despite evidence demonstrating increased protein requirements for optimising muscle mass and functional capacity in older adults [22]. Observational studies have demonstrated that community-dwelling older adults consuming higher protein intakes (1.1 g kg^−1^ day^−1^) lost 40% less lean mass over a 3 year period versus those consuming lower protein intakes (0.7 g kg^−1^ day^−1^) [15]. Further, a 20% higher protein intake was associated with a strong, independent, dose-responsive reduction in risk of incident frailty in older women [23]. Specifically, the PROT-AGE study group recommends average daily protein intake in the range of 1.0 to 1.2 g kg^−1^ day^−1^ to help older adults >65 years maintain and regain lean body mass and function [24].

Despite *evidence that* home-delivery meal interventions in older adults elicit beneficial effects on nutritional status, loneliness, and social well-being [25], Meals on Wheels provision by UK councils have seen a significant decline from 66% in 2014 to just 29% in 2023 [26]. The efficacy of Meals on Wheels has been demonstrated following 12-weeks provision in different populations such as those discharged from hospital [27]. Yet data has largely been generated from studies conducted in the USA where, unlike the UK, there are statutory minimum nutritional requirements for meal delivery provision services [28]. Furthermore, outcome measures in previous research have typically been limited to dietary evaluations via telephone interview with limited clinical, physiological, or psycho-social data collection in the community [28]. There is a need to assess the effect of meal delivery services to improve physical and psychological health in the UK setting. This is pertinent given the increasing prevalence of depression and loneliness amongst community-dwelling older adults [29]. A previous study reported that 3-weeks of daily meal provision to healthy older participants living in the community significantly improved mini nutritional assessment (MNA) score (a validated nutrition screening and assessment tool that can identify older adults who are malnourished or at risk of malnutrition) and self-reported depression score [30]. Further, this study demonstrated the feasibility of physical and psychological health assessments in participants’ homes. Yet, there is a need for more rigorous randomised controlled trials to evaluate the impact of longer-term high protein, high energy home meal delivery services on nutritional status and physical and psychological wellbeing in community dwelling older adults at risk of malnutrition.

An effective high protein, high energy meal delivery service has the potential to improve quality of life, preserve independence and consequently reduce demands upon the health and social care system, if adopted into mainstream local authority or primary care pathways. The aim of this study was to determine whether daily provision of a high energy, high protein meal (>40% daily energy requirements and >50% recommended daily intake for protein) for 12-weeks to under-nourished, community-dwelling, older adults can improve physical, physiological, and psychological outcomes.

## 2 Materials and methods

### 2.1 Study design and randomisation

This randomised crossover trial was designed to determine whether daily provision of a high protein, high energy meal for 12-weeks improved physical and psychological wellbeing. Participants were randomised to complete a 12-week meal intervention block before (meals first group) or after (meals second group) a 12-week period of habitual diet. A stratified randomisation was achieved using the ‘select random sample of cases’ function in IBM SPSS *ver28* whereby participants were stratified by baseline mini nutritional assessment (MNA) score (above and below the median MNA score), and whether they lived alone or cohabited. This ensured an equal number of those cohabiting and with poorer baseline MNA score in each group. This study was approved by the NHS HRA South West - Frenchay Research Ethics Committee (21/SW/0123, 14/10/2021). All participants gave written informed consent to participate, and all work described was carried out in accordance with *The Declaration of Helsinki*.

### 2.2 Recruitment

Community-dwelling older adults were recruited using posters, word of mouth and general practitioner identification of potential participants (using criteria: BMI < 24.5 kg m^−2^ and lived within a 15-mile radius to meal supplier; Dartmoor Community Kitchen Hub (DCKH)) between December 2020 and August 2021. Potential participants were screened via telephone call for initial inclusion criteria; 1) aged ≥ 70 years; 2) no dementia or mental health condition that could severely affect cognitive function; 3) no formal care support or *de facto* resident carer; 4) no hospital overnight stay in the past 3 months; 5) no dysphagia or other conditions limiting the ability to eat (e.g., dental or neurodegenerative disease, masticatory muscle disorders); 6) no cancer drug treatment in the past 6 months; 7) not following a specific prescribed diet. Potential participants were then screened further in their home. Participants who had no severe cognitive impairment (assessed by Adenbrooke’s Cognitive Examination II (score >82) [31] or Montreal Cognitive Assessment (score >17) [32]) and were ‘malnourished’ or ‘at risk of malnutrition’ (as assessed by MNA, score ≤23.5 (33)) were invited to take part in the study.

**Fig. 1:**
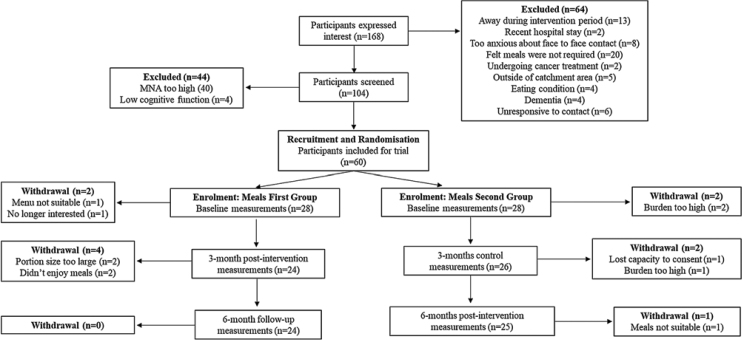
Study flow diagram. MNA; mini nutritional assessment score.

60 participants initially agreed to take part. Forty-nine participants completed the study (16 male, 33 females; 81.8 ± 7.4 years) (Fig. 1). Of the 49 participants who completed the study, 13 (26.5%) cohabited with another person (another participant; *n* = 6; or someone outside the study; *n* = 7) and 36 lived alone. Three participants (6%) were vegetarian. Participants started the research study in 3 batches: March 2022 (*n* = 7), May 2022 (*n* = 19) and August 2022 (*n* = 23).

### 2.3 Meal intervention

During their 12-week intervention period, participants received a daily high protein and high energy meal. Participants selected from a 3-week rolling menu, with 3 options daily. Each high energy and high protein meal was designed to contain at least 50% of daily protein requirements (defined as 1.2 g kg^−1^ day^−1^ for optimal lean body mass and function (24)) for a 70 kg individual. Therefore, each meals provided 0.6 g kg^−1^ or ∼42 g protein per meal. Each meal also contained 40% of daily energy requirements (∼715 kcal for a 70 kg >65 years) [19,20]. On average, the high protein, high energy meals contained 47 ± 7 g of protein and 772 ± 161 kcal energy. For participants >70 kg, to ensure the meals met the recommended nutritional intake, dessert was offered (containing 6 ± 2 g protein and 328 ± 135 kcal). Full nutritional content of meals is shown in Supplementary Table 1. Participants were asked to return any unconsumed food to their delivery driver for weighing of leftovers.

### 2.4 Measurement procedures

Assessment visits took place in the participants’ homes at baseline, following 3-months (post-intervention/post-habitual diet control period), and 6-months (post-intervention/post-habitual diet follow up period). All assessment visits were conducted by the same trained researcher for all participants at all timepoints.

Body weight, height, mid-upper arm circumference and calf circumference were measured. Nutritional status was then assessed using the MNA tool—a validated tool comprising 18 questions related to nutritional status, with a maximum score of 30 [33]. Malnutrition was defined as a score of <17; at risk of malnutrition for scores between 17 and 23.5; and normal nutritional status for scores from ≥24. Resting blood pressure was measured after participants sat quietly for 5 min. Three measurements were taken, 1-min apart and the median value recorded. Handgrip strength was measured using a Jamar handgrip dynamometer according to the Southampton Protocol [34]. Three trials were conducted on each hand and the test hand was alternated. The best score for either hand was used for data analysis. Participants performed the Timed Up and Go (TUG) test whereby they stood up from a standardised chair, walked three meters, turned around, walked back to the chair, and sat down. The time taken to complete the test in seconds was recorded. Walking aids were permitted and mirrored for repeat testing.

Social status was assessed using the MacArthur scale of subjective social status. Measures of psychological and social wellbeing were administered in the order described: Social wellbeing operationalised as loneliness and social capital; The Multidimensional Scale of Perceived Social Support (MSPSS) [35]; Rosenberg Self Esteem (RSE) Questionnaire [36]; Psychological wellbeing assessed as satisfaction with life and mood in the following domains: Happy, proud, relaxed, content, depressed, sad, bored, and stressed; The EQ-VAS and EQ-5D-5L questionnaire for health and an adapted Patient Health Questionnaire-9 depression scale (PHQ-9). Negative mood was calculated by summing the 4 negative mood questions (depressed, sad, bored and stressed).

Nutrient intake was assessed once at each timepoint using an interviewer-administered 24-h dietary recall in line with the multiple-pass approach [37]. Briefly, participants were asked to report all foods and drinks consumed the day prior to the assessment visit (midnight to midnight), followed by further probing on forgotten foods and time of consumption. Further detailed information was gathered on brand names, food preparation methods and condiments used before a final probe. Visual aids were used to portray different portion sizes to enhance reporting accuracy. Energy and macronutrient intakes were quantified using commercially available software; Nutritics diet analysis software [38].

Physical activity was assessed using a wrist-mounted triaxial accelerometer (GENEActive, ActivInsights, Kimbolton, UK) on participant’s non-dominant wrist for 3-days following baseline, 3-months (post-intervention/post-control), and 6-months (post-intervention/follow up) assessment visits [39,40]. Data were extracted using the GENEActive PC Software version 3.3 and processed and analysed using the R-package GGIR (version 2.9-0) [41] in the R environment (version 4.1.2) [42]. We measured physical activity (reported as h per day) using previously validate thresholds (>42.5 mg) on non-dominant wrist worn GENEActives in older adults [43]. When wear time was less than 16 h per 24 h period, data were excluded.

The feasibility of collecting measures of general health, social support, and healthcare costs in this population in participants’ homes was examined. Healthcare and social care resource use was assessed using an adapted version of the healthcare resource use data collection form developed for the NIHR Prevention of Falls Injury Trial [44]. The number of visits to healthcare and social care resources, equipment and aid purchase, healthcare related travel costs and support from relatives or friends in the past 3-months were recorded at each timepoint. Unit costs for each service were taken from PSSRU 2021 [45] or the most recent and available NHS reference costs inflated to 2020/21 prices (Supplementary Table 2). Support costs were calculated based on per hour pay at minimum wage for those >23 years at the time. Travel costs were in line with NHS reimbursement [46].

### 2.5 Blood sampling and analysis

The day following each assessment visit, 26 mL of blood was collected via venepuncture from fasted participants in their homes. Whole blood was immediately analysed for Hb (HemoCue® Hb 201+, HomeCue AB, Angelholm, Sweden). Measurements were repeated 3 times and mean results were reported in g/dL. The remaining blood sample was centrifuged at 4000 rpm for 10-min at 4 °C for preparation of serum which was stored for subsequent analysis at −70 °C. Serum retinol binding protein-4 (RBP4) was assayed in duplicate from serum using a quantitative sandwich enzyme linked immunosorbent assay (ELISA) kit according to manufacturer’s instructions (R&D systems, Biotechne, USA). Sodium, potassium, and chloride were analysed from serum using a Roche Cobas 8000 Ion Selective Electrode modular analyser (Roche, Switzerland). Albumin, ferritin, transferrin, high sensitivity CRP and urea were analysed from serum using a Roche Cobas 8000 c702 clinical chemistry modular analyser using photometric assays (Roche, Switzerland).

#### 2.5.1 Magnetic Resonance Imaging (MRI) analysis

MR data was acquired in self -selected participants with no contraindications to MR scanning (*n* = 15) at baseline, following 3-months (post-intervention/post-habitual diet control period), and 6-months (post-intervention/post-habitual diet follow up period). All MR data acquisition was performed on a Siemens 3.0T MR scanner (MAGNETOM Prisma, Siemens Healthineers, Erlangen, Germany) at the Mireille Gillings NeuroImaging Centre, University of Exeter. Acquisitions were acquired of the upper leg muscles with the body coil positioned such that it was centred mid-thigh. After initial scout images were run to confirm appropriate positioning, a T1-Vibe Dixon anatomical sequence was employed to generate a sequential series of 256 transverse slices which covered the whole area from knee to hip, with an imaging time of 85 s. In-plane resolution was 1.4 × 1.4 mm and a slice thickness of 1.5 mm and a 0.3 mm slice gap. The repetition time (TR) was 11.1 ms with six echo times (TE) acquired ranging from 1.34 to 9.39 ms allowing the generation of separate fat and water images. MRI scans were transferred as DICOM files (Digital Imaging and Communications in Medicine) to open-source medical image processing software 3D Slicer (Version 5.2.1) to allow calculation of fat and muscle volumes. Upper leg scans were manually segmented by only one trained researcher to avoid variability.

### 2.6 Data analysis

#### 2.6.1 Sample size calculation

The sample size calculation was based on the depression score outcome measure, using results from a previous pilot study [30] whereby depression score decreased by an average of approximately 20%

(from 2.21 to 1.79, equivalent to a Cohen’s *D* = mean/SD = 0.42/0.66 = 0.63). A total analysable sample size of *n* = 44 participants would provide 80% power for detecting a moderate to large effect size (Cohen’s *D* = 0.63).

#### 2.6.2 Statistical analysis

The intervention effects for meals first and meals second groups were analysed separately by *t*-test (IBM SPSS ver28) and then pooled using a meta-analysis approach (STATA ver17). The intervention effect for the meals first group was analysed using an independent samples *t*-test comparing baseline to 12-weeks change in the meals first group (meal provision) to the meals second group (consumption of habitual diet). The meals second intervention effect was analysed using a dependent samples *t*-test that compared the 12-weeks to 24-weeks change (meal provision) to the baseline to 12-weeks change (consumption of habitual diet). A pooled intervention effect was achieved by entering the two effects described above into a fixed-effect meta-analysis. The pooled effect was weighted by the sample size (N) to reflect the greater reliability of a same-period control comparison. Intervention effects were expressed in the units of the outcome measure and in standardised units of Cohen’s D with 95%CI, which provides an indication of standardised effect size. *D*∼0.2, ∼0.5 and >∼0.8 were considered small, medium, and large effects respectively.

There were significant increases in protein and energy intake during the habitual diet control period of the meals second group (see results section). This confounded the design principles for a randomised crossover trial resulting in an underestimation of the ‘true’ meal intervention effect. We therefore adapted our statistical analysis approach to conduct post-hoc analysis of the pre–post meal intervention data via paired *t*-test for the meals first (baseline to 12 weeks change) and meals second (12 weeks to 24 weeks change) groups. The pooled effects were calculated via meta-analysis. Levene’s test was used to check for the assumption of equality of variances for the independent samples *t*-tests.

## 3 Results

Baseline characteristics are shown in Table 1. From a target of 84 meals over 12 weeks, participants received 80 ± 7 meals. After taking leftovers into account, participants consumed approximately 783 ± 156 kcal day^−1^ and 43 ± 6 g protein, which was the equivalent of 0.65 ± 0.14 g kg^−1^ day^−1^ per meal.

**Table 1:** Participant baseline characteristics.

	Total (*n* = 56)	Meals first condition (*n* = 25)	Meals second condition (*n* = 24)
Sex (m:f)	17:39	9:16	7:17
Live Alone:Cohabit	40:16	17:8	16:8
Age (years)	82 ± 7	81 ± 7	82 ± 8
BMI (kg m^−2^)	26.6 ± 5.4 (*n* = 53)	25.3 ± 4.5 (*n* = 23)	27.4 ± 4.7 (*n* = 24)
Systolic Blood Pressure (mmHg)	137 ± 20	133 ± 24	142 ± 17
Diastolic Blood Pressure (mmHg)	81 ± 12	78 ± 14	84 ± 9
Timed up and go (s)	16.1 ± 8.9 (*n* = 51)	15.5 ± 7.5 (*n* = 23)	17.0 ± 10.5 (*n* = 23)
Hand grip strength (kg force)	18 ± 10 (*n* = 53)	20 ± 9 (*n* = 24)	18 ± 13 (*n* = 24)
Calf circumference (cm)	34.9 ± 4.0	34.1 ± 4.6	35.5 ± 3.5
Mid upper arm circumference (cm)	27.5 ± 4.5	27.3 ± 4.4	27.6 ± 3.9
Physical activity (hours/day)	3.0 ± 1.6	3.1 ± 1.4	2.9 ± 1.7
Haemoglobin (g/dL)	13.4 ± 1.8 (*n* = 42)	13.5 ± 2.0 (*n* = 19)	13.5 ± 1.6 (*n* = 20)
Nutritional Assessment MNA (/30)	21.0 ± 2.5	21.0 ± 2.5	21.2 ± 1.7
Subjective social status	6 ± 2	6 ± 2	6 ± 2
Social support (MDPSS /84)	62 ± 16	58 ± 18	63 ± 14
Self-esteem (RSE /30)	20 ± 5	20 ± 6	20 ± 6
Modified PHQ (/24)	7 ± 5	6 ± 5	7 ± 6
Healthcare costs (£ in last 3 months)	866 ± 1338	820 ± 1356	910 ± 1343
Support costs (£ per week)	214 ± 168	243 ± 156	188 ± 177

Data are presented as means ± standard deviation. M; males. F; females. BMI; body mass index. Kg; kilogram. M; metres. mmHg; millimetres of mercury. S; seconds. CC; calf circumference. MUAC; mid-upper arm circumference. Cm; centimetre. Hb; haemoglobin. G; g. dL; decilitre. MNA; mini nutritional assessment score. MDPSS; multidimensional perceived social support scale. RSE; Rosenberg’s self-esteem scale. PHQ; patient health questionnaire.

### 3.1 Estimated average effect of the intervention

There was no significant intervention effect on depression measured via PHQ, or on loneliness (Table 2). There was no significant intervention effect on quality of life, satisfaction with life, perceived social support, or perceived social status (Supplementary Table 3). Overall, there was a moderate pooled effect on negative mood, showing it was reduced over time (pooled *D* = −0.56, *p* < 0.01) (Table 2).

**Table 2:** Intervention effect on measures of psychological wellbeing, nutritional status, body composition and physical function. Pooled intervention effects are presented in bold and were calculated by entering the meals first and meals second group effects into a fixed-effect meta-analysis.

	Comparison	Intervention effect		
		Mean difference	Cohen’s D (95% CI)	P value
Psychological wellbeing				
Depression (PHQ av8) score	Meals first intervention effect (*n* = 49)	−0.08	−0.19 (−0.75 to 0.37)	0.51
	Meals second intervention effect (*n* = 24)	−0.04	0.05 (−0.36 to 0.45)	0.82
	**Pooled effect**	**−0.07**	**−0.11 (−0.51 to 0.29)**	**0.59**
Negative mood score	Meals first intervention effect (*n* = 49)	−2.34	−0.65 (−1.22 to −0.07)	0.03*
	Meals second intervention effect (*n* = 24)	−2.62	−0.37 (−0.77 to 0.05)	0.09
	**Pooled effect**	**−2.43**	**−0.56 (−0.97 to −0.15)**	<0.01*
Loneliness score	Meals first intervention effect (*n* = 49)	−0.25	−0.11 (−0.67 to 0.45)	0.70
	Meals second intervention effect (*n* = 24)	−0.96	−0.24 (−0.64 to 0.17)	0.25
	**Pooled effect**	**−0.48**	**−0.45 (−098 5 to 0.07)**	**0.46**
Nutritional status				
Nutritional status (MNA score)	Meals first intervention effect (n = 49)	−1.87	0.85 (0.26–1.43)	<0.01*
	Meals second intervention effect (n = 24)	2.06	0.50 (0.07 to 0.92)	<0.02*
	**Pooled effect**	**1.93**	**0.74 (0.32–1.15)**	<0.001*
Energy Intake (kcal day^−1^)	Meals first intervention effect (*n* = 47)	48.5	0.09 (−0.49 to −0.66)	0.77
	Meals second intervention effect (*n* = 23)	63.7	0.06 (−0.35 to 0.47)	0.76
	**Pooled effect**	**53.5**	**0.08 (−0.33 to 0.49)**	**0.70**
Protein intake (g. kg^−1^ day^−1^)	Meals first intervention effect (*n* = 45)	0.12	0.29 (−0.30 to 0.88)	0.33
	Meals second intervention effect (*n* = 23)	−0.02	−0.03 (−0.44 to 0.38)	0.90
	**Pooled effect**	**0.07**	**0.18 (−0.23 to 0.60)**	**0.39**
Body composition				
BMI (kg m^−2^)	Meals first intervention effect (*n* = 47)	−0.03	−0.03 (−0.60 to 0.55)	0.93
	Meals second intervention effect (*n* = 24)	0.14	0.10 (−0.30 to 0.50)	0.63
	**Pooled effect**	**0.03**	**0.01 (−0.39 to 0.42)**	**0.95**
Mid-thigh muscle volume (cm^−3^)	Meals first intervention effect (*n* = 7)	−0.92	−1.25 (−2.35 to −0.11)	0.03*
	Meals second intervention effect (*n* = 8)	−0.07	−0.09 (−0.78 to 0.61)	0.82
	**Pooled effect**	**−0.61**	**−0.83 (−1.58 to 0.07)**	0.03*
Physical function				
Handgrip strength (kg)	Meals first intervention effect (*n* = 45)	−0.96	0.30 (−0.29 to 0.89)	0.32
	Meals second intervention effect (n = 21)	0.48	0.10 (−0.33 to 0.53)	0.65
	**Pooled effect**	**0.81**	**0.24 (−0.19 to 0.66)**	**0.28**
Physical activity (hours/day)	Meals first intervention effect (*n* = 45)	0.33	0.41 (−0.19 to 1.00)	0.18
	Meals second intervention effect (*n* = 21)	0.35	0.23 (−0.21 to 0.66)	0.31
	**Pooled effect**	**0.34**	**0.35 (−0.08 to 0.78)**	**0.11**
Systolic blood pressure (mmHg)	Meals first intervention effect (*n* = 49)	7.50	0.42 (−0.15 to 0.99)	0.15
	Meals second intervention effect (*n* = 24)	7.96	0.32 (−0.09 to 0.73)	0.13
	**Pooled effect**	**7.65**	**0.39 (−0.02 to 0.79)**	**0.06**
Diastolic blood pressure (mmHg)	Meals first intervention effect (*n* = 49)	5.71	0.55 (−0.02 to 1.12)	0.06
	Meals second intervention effect (*n* = 24)	7.30	−0.37 (−0.04 to 0.79)	0.08
	**Pooled effect**	**6.23**	**0.49 (0.09 to 0.90),**	0.017*

Data are presented as means. Statistical significance accepted as *p < 0.05. Between group comparison between meals first group and meals second group from week 0–12 whereby the meals first group received the meal provision intervention and meals second group did not (consumption of habitual diet). Within group comparison in meals second group only comparing week 0–12 (consumption of habitual diet) vs week 12–24 (whereby they did receive the meal provision intervention). PHQ av8; patient health questionnaire score, average. MNA; mini nutritional assessment. Kcal; kilocalories. G; grams. Kg; kilograms. BMI; body mass index. Kg; kilograms. M; metres. Cm; centimetres. Kg; kilograms. Sec; seconds.

The meal intervention significantly increased MNA score with a medium effect size (MNA: pooled D = 0.74, *p* < 0.001). There was no significant effect on energy intake (energy intake: pooled *D* = 0.08, *p* = 0.70) or protein intake, (pooled *D* = 0.18, *p* = 0.39) (Table 2).

There was no significant effect of meal intervention on BMI (pooled *D* = 0.01, *p* = 0.95), CC (pooled *D* = −0.12, *p* = 0.56), or MUAC (pooled *D* = 0.07, *p* = 0.72) (Table 2). Muscle volume was assessed in a subset of 15 participants and increased during the intervention for 9 participants, was unchanged in 2 participants, but decreased in 4 participants. There were individual circumstances of ill-health in the 4 participants who lost muscle volume (pooled *D* = −0.83 (−1.58 to −0.07, *p* = 0.03).

There was no significant effect of meal intervention on handgrip strength (pooled *D* = 0.81, *p* = 0.28), physical activity (pooled *D* = 0.34, *p* = 0.11) or systolic blood pressure (pooled *D* = 0.39, *p* = 0.06) (Table 2). Diastolic blood pressure significantly increased post-intervention (pooled *D* = 0.49, *p* = 0.02).

There was no significant effect of meal intervention on any blood biomarkers including CRP (pooled *D* = 1.06, *p* = 0.89), Hb (pooled D = 0.48, *p* = 0.054), or RBP4 (pooled *D* = 0.12, *p* = 0.63) (Supplementary Table 3.). Nor was there any effect on serum sodium, potassium, chloride, albumin, ferritin and transferrin and analytes were within the normal population range (Supplementary Table 3.).

### 3.2 Feasibility

All appointments with participants went ahead as scheduled. Tests were safely conducted in participants’ homes using portable equipment. Height and weight were deemed safe to measure in 93.9% of participants. Handgrip strength data were collected in 89.8% participants (not available for *n* = 5 due to arthritic wrist pain). The TUG test was deemed safe to conduct in 91.8% of participants. Blood samples were collected from 85.7% participants (was not collected from *n* = 6 due to difficulty accessing veins and *n* = 1 needle related anxiety). Questions relating to quality of life, loneliness, mood, and self-esteem were completed by 95.9% participants. Measures of general health, social support and healthcare costs were successfully collected in participant homes at 12-week intervals demonstrating feasibility of collection and analysis of these measures.

#### 3.2.1 Post-hoc analysis: effect of meal intervention using within group pre-to-post changes only

Energy and protein intake increased significantly during the control period where participants were asked to maintain their habitual diet in the meals second group (Energy intake: increase = 252 kcal [95% CI 36–487 kcal], t(22) = 2.408, *p* = 0.025, Protein intake: increase = 0.20 g kg^−1^ [95% CI 0.04–0.357 g kg^−1^], t(22) = 2.629, *p* = 0.015), which confounded the principle of a randomised crossover design. Given that these effects could contribute to an underestimation of the ‘true’ meal intervention effect, post-hoc analysis was performed to test the changes before and after the 12-week meal intervention in the meals first and meals second group and then pooled using meta-analysis. This analysis was undertaken for a sub-set of outcome measures, selected in a hypothesis-driven fashion (protein and energy intakes, nutrition assessment, grip strength, depression, negative mood scores and mid-thigh muscle volume).

**Fig. 2:**
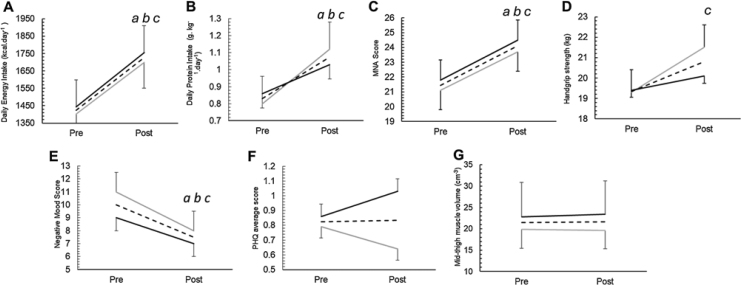
Changes in dietary intake (A. Energy intake, B. Protein intake C. MNA score, D. Handgrip strength, E. Negative Mood score F. PHQ average score and G. Mid-thigh muscle volume) pre-post intervention in meals first group, meals second group, and combined effect. Data presented as means ± SEM. a; *p* < 0.05 in meals first group. b; *p* < 0.05 in meals second group, c; *p* < 0.05 average effect. Kcal; kilocalories, g; grams, kg; kilograms, PHQ; patient health questionnaire.

For the most part this post-hoc analysis revealed larger and more statistically significant intervention effects than those of the crossover analysis. Daily energy intake increased by 311 kcal (*D* = 0.52, *p* < 0.001) and protein intake increased by 0.24 g kg^−1^ (*D* = 0.50, *p* < 0.01) during the 12-week meal periods indicating the intervention induced an improvement of moderate effect size (Fig. 2.). Furthermore, post hoc analysis revealed MNA score (2.6 points, large effect size, *D* = 1.14 (0.78–1.50), *p* < 0.001), and handgrip strength significantly improved (1.5 kg, small/medium effect size, *D* = 0.36 (0.06–0.66), *p* = 0.02). No significant changes were observed in levels of depression (PHQ average, *D* = −0.20 (−0.48 to 0.09), *p* = 0.17) but a significant reduction was observed in negative mood (−2.6 points, *D* = −0.59 (−0.88 to 0.09), *p* < 0.001) (Fig. 2.). No significant changes were observed in mid-thigh muscle volume *D* = 0.40 (−0.21 to 1.0), *p* = 0.20

#### 3.2.2 Retention of interventional changes in the meals first group

The retention of the favourable effect of the meal intervention was assessed in the meals first group only. Twelve weeks after meal removal, the following % of the meal effect was retained: 68% of the improvements in MNA score were remaining, 27% for negative mood score, 15% for daily energy intake, 6% for daily protein intake and 0% for handgrip strength.

## 4 Discussion

Community-dwelling older adults are at risk of malnutrition, and their protein intake may be suboptimal [5,6]. In the present study provision of a daily high protein, high energy meal (providing >40% daily energy requirements and >50% recommended daily intake for protein) for 12-weeks to under-nourished older adults living independently in the community improved dietary protein and energy intake, nutritional status, and aspects of physical function. However, these benefits were not retained over a 12-week follow up period after removal of the meals, indicating the need for a continued intervention in this population.

The high protein, high energy meal delivery service significantly increased daily energy intake (from 1423 ± 533 kcal day^−1^ to 1728 ± 480 kcal.day^−1^) and protein intake (from 0.83 ± 0.33 g kg^−1^ day^−1^ to 1.08 ± 0.45 g kg^−1^ day^−1^) with a large and medium effect size respectively. This resulted in significantly improved nutritional status, such that 55% of participants moved from sub-optimal nutrition status, to ‘no risk of malnutrition’ status. However, participants did not achieve the recommended daily dietary requirements of 1788 kcal day^−1^ and 1.2 g kg^−1^ day^−1^ [19,20]. This is despite, the intervention meals providing 47 ± 7 g of protein and 772 ± 161 kcal energy intake with an additional 6 ± 2 g protein and 328 ± 135 kcal for those >70 kg to meet 50% daily protein requirements (0.6 g kg^−1^ or ∼42 g protein per meal) and 40% daily energy requirements (∼715 kcal for a 70 kg >65 years). This may be because participants were unable to consume *all* of the provided meal, since it is well reported that the appetite of under-nourished older adults is supressed [12]. However, after consideration of leftovers, the measured consumption of meals was 783 ± 156 kcal day^−1^ and 43 ± 6 g protein (0.65 ± 0.14 g kg^−1^ day^−1^). It therefore seems more likely that food consumption in the other daily meals was attenuated, and so recommended daily intake was not achieved. Given the intervention increased daily protein intake to 1.08 ± 0.45 g kg^−1^ day^−1^ and the consumed intervention meal provided 0.63 ± 0.15 g kg^−1^ day^−1^, the remaining 0.45 g kg^−1^ day^−1^ was consumed over the further 2 daily meals (∼26 g/0.23 g kg^−1^ per meal). The per-meal anabolic threshold of dietary protein intake in older individuals to maintain lean mass and optimal function is 25–30 g protein per meal [24]. Whilst the daily intervention meal exceeded this threshold, it is highly unlikely that the other daily meals (breakfast and dinner meals) consumed were meeting the anabolic threshold for this population, highlighting the need for future meal provision services to be designed to ensure anabolic stimulation is achieved throughout the day i.e., through provision of a high protein meal and snack. Following removal of the intervention for 12-weeks (meals first group), the gains previously achieved in energy and protein intake were substantially depleted (by 85% and 94% respectively towards baseline values). The improvements in handgrip strength had also disappeared suggesting functional benefits are also dependent on continued intervention.

The observed beneficial effects of home meal delivery on dietary intake, nutritional risk and quality of life are consistent with other studies of meal delivery amongst older adults. Previously, following a shorter duration 3-week daily meal provision service (3 meals per day) in 19 older adults (8M/11F; 78.3 ± 8.7 years) participants rated themselves as significantly less depressed (*p* = 0.03) [30] as assessed via 1-item, using a Likert-type response scale. Fewer depressive symptoms (*p* < 0.05) were also observed when using The Geriatric Depression Scale (Short Form) following a breakfast intervention in older adults >60 years [47]. Whilst in the current study we observed reductions in negative mood, we did not observe changes in levels of depression. This is perhaps unsurprising given that to isolate the intervention effects to the *consumption* of the provided meals and to ensure consistency across participants, the meal deliverers were instructed to minimise their interactions with the participants. This contrasts with previously published studies where such controls on social interactions with participants were not in place [48]. Thus, providing confidence that the social interaction involved in prior studies contributed to participants becoming less depressed after the meal delivery service and highlighting the importance of these interactions for overall wellbeing. Certainly the social interaction provided by a meal service has previously been shown to lessen feelings of isolation and loneliness and would likely contribute positively to the beneficial effects on psychological wellbeing [49].

Here, we demonstrated a significant increase in overall MNA score with a large effect size. Eighty-six percent of participants had an improvement in MNA score, with 55% of participants moving from sub-optimal nutrition, to above the ‘no risk of malnutrition’ threshold. This is consistent with previous research that demonstrated there was a significant improvement in nutritional status as assessed by the MNA short form following two months of home-delivered meals (3 meals per week) in 66 US based older adults >55 years [50]. This was in line with an average energy intake of 403 kcal and 21 g of protein. Notably, the large improvement in MNA score in the current study was observed in participants who on average, had MNA scores within the ‘at risk of malnutrition’ threshold at baseline (21.0 ± 2.5). Previous research has demonstrated MNA scores have still significantly improved in older adults following meal delivery interventions - even when baseline MNA score have been above the well-nourished threshold at baseline (24.3 ± 2.8) [30]. This demonstrates the all-encompassing benefits of meal delivery services for *all* older adults and potentially increased benefits for those who have lower malnutrition scores (MNA score <17). We demonstrated in the meals first group (*n* = 25) that 12-weeks following removal of the meal delivery service, there was a 32% decline in MNA score towards initial baseline values with a return, on average to the ‘at risk of malnutrition’ category (22.9 ± 2.7). MNA score is a good prognostic indicator of frailty [51], functional decline [52], and mortality [52] in older adult populations, and so confirms the need for a continued intervention to retain these important benefits.

Handgrip strength is often used as an indicator of general muscle strength in older adults, with weak handgrip strength associated with reduced lean body mass [53] and poorer physical performance [54]. Indeed, it is included as a parameter in many tools used to diagnose sarcopenia [55,56]. In the current study, daily meal provision significantly increased handgrip strength by a small/medium effect size from 19.3 ± 10.9 kg to 20.9 ± 11.1 kg. Improvement in handgrip strength is likely explained by the improvements in nutritional status and dietary intake elicited by the meal delivery intervention. Previous protein intervention studies have reported 12-weeks of protein supplementation significantly improved handgrip strength of community-dwelling older adults (28.7 ± 10.1 kg to 29.6 ± 10.0 kg) [57]. It is encouraging that similar findings are observed for community-dwelling older adults in the current study. Yet, meal delivery interventions need to be sustained as we demonstrated in the meals first group, when participants returned to habitual diet for 12-weeks following the meal provision intervention, handgrip strength had returned to original baseline values. MRI scans were obtained in a subset of participants (*n* = 15) where it was safe to do so. Initial statistical analysis demonstrated a significant decline in muscle volume following the intervention in this subset of participants, yet this effect was lost on secondary analysis. Given the small sample size included for MRI analysis, these results must be treated with caution as analyses are vulnerable to individual changes caused by specific frailty or medical concerns which are prevalent in this population.

Older adults often have a range of comorbidities, acute periods of illness, and periods of hospitalisation—all of which may contribute to malnutrition risk. The use of 24 h dietary recall to assess dietary intake has previously been validated, but a key limitation is that it captures only a single day and as such cannot account for day-to-day variation in diet [58]. The 24 h dietary recall method was used for dietary intake assessment given its low participant burden compared with diet diaries, but a heavier reliance on short term memory [59]. The 24 h recall method was feasible within this cohort who had no severe cognitive impairments; however, this should be reconsidered when conducting similar measurements in those with cognitive decline or dementia. This study was designed as a randomised, crossover design to allow all recruited participants to benefit from the meal provision, whilst also gathering control data. A valuable, but challenging observation in the current study was that energy and protein intake increased significantly from baseline during the 12-week pre intervention habitual diet control period in the meals second group. This increase in energy and protein intake during the habitual diet control period could be attributed to two plausible and related explanations: (i) a ‘hawthorn effect’ – whereby participants improved their eating habits since they were aware that this was being measured and/or, (ii) participants improved their eating habits due to the knowledge acquired of the meals they would receive in the next phase of the study i.e. high energy and high protein. Whilst findings from such population-based’ real world’ studies are ecologically valid and translatable, these observations highlight the difficulties of designing and conducting a traditional randomised controlled trial within ecologically valid, population-based settings. This confounding of the design principles for a randomised crossover trial also necessitated a change in the statistical approach due to the potential for underestimation of the ‘true’ meal intervention effect. Another intrinsic study limitation was that neither participants nor the researchers could be blinded due to the characteristics of the meal intervention.

Here, we have demonstrated the feasibility of carrying out an expanded range of physiological measures (e.g. BMI, handgrip strength, tests of mobility), psychological measures (e.g., depression, self-esteem, loneliness) and more invasive measures such as blood sampling via venepuncture in participants’ homes with the appropriate planning, as well as assessing healthcare resource use to estimate associated costs. Future research in larger scale studies should incorporate such measurement techniques to allow for economic evaluations of interventions including validated costing estimates.

In line with previous research [60], this study highlighted that the optimal protein requirements for older adults (1.2 g kg^−1^ day^−1^) are difficult to meet. Anecdotally, participants reported that the volume of the provided meals (which was required to achieve nutritional targets) was unappealing, leading to some (measured) plate waste. High protein meals need to be appealing and easy to eat, since for such public health interventions to be effective, meals must be consumed and without attenuating nutrient intake from other meals. Whilst the current study was too small and short term to measure cost effectiveness, the feasibility of quantifying cost effectiveness has been demonstrated. A larger scale, multi-centre longer duration study is now required to assess Meals on Wheels cost effectiveness, with the goal of supporting translation into a national programme.

## 5 Conclusions

Provision of high protein, high energy meals to community-dwelling older adults for 12-weeks significantly increased daily energy and protein intake by 21.4 and 30.1% respectively. Consequently nutritional status and handgrip strength increased significantly, indicative of reduced risk of frailty [51], functional decline and mortality [52]. However, daily energy and protein intake, nutritional status and handgrip strength returned towards or to baseline on removal of the meal provision, reinforcing the need for sustained intervention. Thus, home-delivered meals offer a scalable intervention for community-dwelling older adults to prevent malnutrition, promote health and sustain high quality independent living, thus reducing the burden of ageing and frailty on health and social care systems.

## Funding source

Torbay Medical Research Fund, Hadley Trust, Devon County Council, University of Exeter, and Plymouth University. The funders had no input in study design; in the collection, analysis and interpretation of data; in the writing of the report; or in the decision to submit the article for publication.

## Declaration of competing interest

Prof Joanna Bowtell reports financial support for the provision of meals was provided by The Hadley Trust and Devon County Council. Prof Joanna Bowtell reports a research grant was awarded by Torbay Medical Research Fund. If there are other authors, they declare that they have no known competing financial interests or personal relationships that could have appeared to influence the work reported in this paper.

## CRediT authorship contribution statement

**Lauren Struszczak:** Data curation, Formal analysis, Investigation, Project administration, Visualization, Writing - original draft. **Mary Hickson:** Conceptualization, Funding acquisition, Methodology, Writing - review & editing. **Irene McClelland:** Conceptualization, Funding acquisition, Methodology, Writing - review & editing. **Brad Metcalf:** Conceptualization, Funding acquisition, Formal analysis, Methodology, Writing - original draft. **Manuela Barreto:** Conceptualization, Funding acquisition, Methodology, Writing - review & editing. **Luciana Torquati:** Conceptualization, Funding acquisition, Methodology, Writing - review & editing. **Jon Fulford:** Data curation, Methodology, Writing - review & editing. **Rachael Allen:** Data curation, Writing - review & editing. **Claire Hulme:** Conceptualization, Funding acquisition, Methodology, Writing - review & editing. **Mary F. O’Leary:** Conceptualization, Funding acquisition, Writing - review & editing. **Joanna L. Bowtell:** Conceptualization, Funding acquisition, Methodology, Writing - original draft.
