# Supplementary material for: Provision of a daily high protein and high energy meal: Effects on the physical and psychological wellbeing of community-dwelling, malnourished older adults; a randomised crossover trial
Source: J Nutr Health Aging. 2024 Dec 10;29(2):100429. doi: 10.1016/j.jnha.2024.100429 (PMC12180064; doi:10.1016/j.jnha.2024.100429)
Supplement: Supplementary file 1 [file mmc1.docx]

**Supplementary Materials**

**Supplementary Table 1. Nutritional content of intervention meals**

|  | **Energy (kcal)** | **Protein (g)** | **Fat (g)** | **Saturated Fat (g)** | **Carbohydrates (g)** | **Vitamin D (μg)** | **B12 (μg)** | **Calcium (mg)** |
| --- | --- | --- | --- | --- | --- | --- | --- | --- |
| **Omnivore meals (n=31)** | 769 ± 161 | 49 ± 9 | 42 ± 13 | 21 ± 8 | 47 ± 16 | 0.7 ± 0.8 | 1.2 ± 1.4 | 187.5 ± 95.5 |
| **Vegetarian meals (n=10)** | 905 ± 154 | 45 ± 5 | 52 ± 13 | 25 ± 6 | 59 ± 22 | 0.6 ± 0.6 | 0.9 ± 0.7 | 335.0 ± 267.8 |
| **Desserts (n=7)** | 328 ± 135 | 6 ± 2 | 17 ± 6 | 9 ± 4 | 39 ± 19 | 0.8 ± 0.5 | 0.6 ± 0.1 | 82.9 ± 28.8 |

Kcal; kilocalories. g; grams. μg; microgram. Mg; milligram.

**Supplementary Table 2. Unit costs of healthcare resource use items price year 2020/21**

| *Community Healthcare Service* | Location | Unit cost |
| --- | --- | --- |
| General Practitioner surgery | Clinic | £39.23^1^ |
|  | Home visit | £117.69^1^ |
| Practice Nurse | Clinic | £10.85^1^ |
| Community Nurse | Home visit | £19.25^1^ |
| Physiotherapist | Clinic | £103.54^1^ |
| Chiropodist | Clinic | £41.00^1^ |
| Speech and language therapy | Clinic | £126.91^2^ |
| Dietetics | Clinic | £97.33^2^ |
| Counsellor | Clinic | £51.00^1^ |
| Social worker | Home visit | £46.00^1^ |
| Home help or care worker | Home visit | £12.27^1^ |
| Dentist | Clinic | £44.33^1^ |
| Podiatrist | Clinic | £27^1^ |
| Audiologist | Clinic | £140^1^ |
| Exercise/balance class | Clinic | £67^1^ |
| Osteopathy | Clinic | £27.00^1^ |
| Optician | Clinic | £117^1^ |
| Reflexology | Private clinic | £45^3^ |
| Chiropractor | Private clinic | £42^4^ |
| *Hospital services* |  | **Unit cost** |
| Inpatient stay (24 hours) |  | £602.52^5^ |
| Hospital day centre |  | £361.81^2^ |
| Outpatient visit |  | £142.58^5^ |
| A & E |  | £481.19^2^ |
| Ambulance |  | £134^1^ |
| Nursing /residential home |  | £870^1^ |
| *Support* |  | £9.50 per hour^6^ |

^1^PSSRU 2021, ^2^ NHS Reference costs 2020/21, ^3^taken from Exeter Reflexology, ^4^taken from South West costs, ^5^NHS Reference costs 2017/18 inflated to 2020/21 using 2.21%(45), ^6^ based on minimum wage for >23 year olds 2020/21.

**Supplementary Table 3. Intervention effect on measures of psychological wellbeing, social status and social support, body composition, physical function and blood biomarkers.**

|  | **Comparison** | **Intervention Effect** | | |  |
| --- | --- | --- | --- | --- | --- |
|  |  | **Mean difference** | **Cohen’s D (95% CI)** | **P value** |  |
| **Psychological wellbeing, social status and social support** |  |  |  |  |  |
| ***Self-Esteem (RSE) Score*** | Meals first intervention effect (n=48) | 1.0 | 0.25 (-0.32 to 0.82) | 0.39 |  |
|  | Meals second intervention effect (n=22) | 0.6 | 0.07 (-0.35 to 0.49) | 0.74 | |
|  | **Pooled effect** | **0.87** | **0.19 (-0.22 to 0.61)** | **0.35** | |
| ***Satisfaction Score*** | Meals first intervention effect (n=48) | 2.33 | 0.19 (-0.38 to -0.76) | 0.52 | |
|  | Meals second intervention effect (n=23) | -0.09 | -0.18 (-0.43 to 0.39) | 0.93 | |
|  | **Pooled effect** | **1.55** | **0.12 (-0.29 to -0.53)** | **0.56** | |
| ***Perceived Social Support (MDPSS)*** | Meals first intervention effect (n=48) | 5.77 | 0.35 (-0.22 to 0.92) | 0.23 | |
|  | Meals second intervention effect (n=22) | 6.09 | 0.25 (-0.18 to -0.67) | 0.26 | |
|  | **Pooled effect** | **5.87** | **0.32 (-0.09 to 0.73)** | **0.12** | |
| ***Subjective Health (EQ5D VAS)*** | Meals first intervention effect (n=49) | 3.19 | 0.17 (-0.39 to -0.74) | 0.55 | |
|  | Meals second intervention effect (n=24) | -2.50 | -0.08 (-0.48 to 0.32) | 0.70 |  |
|  | **Pooled effect** | **1.32** | **0.09 (-0.31 to 0.49)** | **0.66** |  |
| ***Quality of Life (EQ5D5L)*** | Meals first intervention effect (n=49) | 0.46 | 0.12 (-0.44 to 0.68) | 0.68 |  |
|  | Meals second intervention effect (n=24) | -0.04 | -0.01 (-0.41 to 0.39) | 0.96 |  |
|  | **Pooled effect** | **0.30** | **0.08 (-0.32 to 0.47)** | **0.66** |  |
| **Body Composition** |  |  |  |  |  |
| ***Calf circumference***  ***(cm)*** | Meals first intervention effect (n=49) | -0.51 | -0.32 (-0.89 to 0.24) | 0.93 |  |
|  | Meals second intervention effect (n=24) | 0.43 | 0.29 (-0.12 to 0.70) | 0.63 |  |
|  | **Pooled effect** | **-0.02** | **-0.12 (-0.52 to 0.28)** | **0.56** |  |
| ***Mid upper-arm circumference***  ***(cm)*** | Meals first intervention effect (n=48) | 0.13 | 0.07 (-0.49 to 0.64) | 0.27 |  |
|  | Meals second intervention effect (n=24) | 0.16 | 0.08 (-0.78 to 0.61) | 0.17 |  |
|  | **Pooled effect** | **0.14** | **0.07 (-0.33 to 0.47)** | **0.72** |  |
| **Physical function** |  |  |  |  |  |
| ***Timed up and go***  ***(sec)*** | Meals first intervention effect (n=45) | 1.09 | 0.28 (-0.31 to 0.86) | 0.36 |  |
|  | Meals second intervention effect (n=22) | 0.83 | 0.10 (-0.32 to -0.52) | 0.64 |  |
|  | **Pooled effect** | **1.00** | **0.32 (-0.20 to 0.64)** | **0.30** |  |
| **Blood biomarkers** |  |  |  |  |  |
| ***CRP (mg/L)*** | Meals first intervention effect (n=45) | -0.57 | -0.15 (-0.83 to 0.53) | 0.68 |  |
|  | Meals second intervention effect (n=21) | 4.41 | 0.41 (-0.10 to 0.92) | 0.12 |  |
|  | **Pooled effect** | **1.06** | **0.03 (-0.45 to 0.52)** | **0.67** |  |
| ***Hb (g/dL)*** | Meals first intervention effect (n=45) | 0.83 | 0.72 (0.03 to 1.40) | 0.04* |  |
|  | Meals second intervention effect (n=22) | -0.05 | -0.02 (-0.50 to 0.46) | 0.94 |  |
|  | **Pooled effect** | **0.54** | **0.48 (-0.01 to 0.97)** | **0.05** |  |
| ***Ferritin (µg/L)*** | Meals first intervention effect (n=33) | -10.23 | -0.38 (-1.97 to -0.31) | 0.28 |  |
|  | Meals second intervention effect (n=16) | 8.18 | 0.07 (-0.42 to 0.56) | 0.78 |  |
|  | **Pooled effect** | **-4.22** | **-0.23 (-0.73 to -0.26)** | **0.35** |  |
| ***Transferrin (µmol/L)*** | Meals first intervention effect (n=33) | -1.06 | -0.49 (-1.2 to 0.23) | 0.18 |  |
|  | Meals second intervention effect (n=14) | -1.38 | -0.37 (-0.91 to -0.18) | 0.19 |  |
|  | **Pooled effect** | **-1.16** | **-0.45 (-0.98 to 0.07)** | **0.09** |  |
| ***Albumin (g/L)*** | Meals first intervention effect (n=34) | -0.65 | -0.36 (-1.0 to 0.32) | 0.30 |  |
|  | Meals second intervention effect (n=14) | -2.19 | -0.68 (-1.22 to -0.13) | 0.02* |  |
|  | **Pooled effect** | **-0.14** | **-0.47 (-0.96 to 0.03)** | **0.06** |  |
| ***Chloride(mmol/L)*** | Meals first intervention effect (n=34) | 1.29 | 0.64 (-0.06 to 1.32) | 0.07 |  |
|  | Meals second intervention effect (n=16) | -0.19 | -0.19 (-0.69 to -0.30) | 0.45 |  |
|  | **Pooled effect** | **0.82** | **0.37 (-0.12 to 0.89)** | **0.14** |  |
| ***Potassium(mmol/L)*** | Meals first intervention effect (n=32) | -0.12 | -0.26 (-0.96 to 0.45) | 0.48 |  |
|  | Meals second intervention effect (n=16) | -0.06 | -0.10 (-0.63 to 0.43) | 0.71 |  |
|  | **Pooled effect** | **-0.10** | **-0.21 (-0.72 to 0.31)** | **0.43** |  |
| ***Sodium (mmol/L)*** | Meals first intervention effect (n=34) | 1.35 | 0.48 (-0.21 to 1.16) | 0.17 |  |
|  | Meals second intervention effect (n=16) | -0.31 | -0.07 (-0.55 to 0.43) | 0.80 |  |
|  | **Pooled effect** | **0.82** | **0.31 (-0.18 to 0.80)** | **0.22** |  |
| ***Urea (mmol/L)*** | Meals first intervention effect (n=34) | 0.34 | 0.19 (-0.49 to -0.86) | 0.59 |  |
|  | Meals second intervention effect (n=16) | 0.71 | 0.32 (-0.19 to 0.81) | 0.23 |  |
|  | **Pooled effect** | **0.46** | **0.23 (-0.26 to -0.71)** | **0.36** |  |
| ***RBP4 (mg/L)*** | Meals first intervention effect (n=34) | -2.6 | -0.07 (-0.74 to 0.60) | 0.08 |  |
|  | Meals second intervention effect (n=16) | 36.4 | 0.52 (-0.02 to 1.03) | 0.06 |  |
|  | **Pooled effect** | **9.9** | **0.12 (-0.37 to 0.60)** | **0.63** |  |

Data are presented as means. Statistical significance accepted as *p<0.05. Between group comparison between meals first group and meals second group from week 0-12 whereby the meals first group received the meal provision intervention and meals second group did not (consumption of habitual diet). Within group comparison in meals second group only comparing week 0-12 (consumption of habitual diet) vs week 12-24 (whereby they did receive the meal provision intervention). *RSE; Rosenberg’s self-esteem score. MDPSS; multidimensional scale of perceived social support. VAS; visual analogue scale*. *CRP; CRP. Hb; haemoglobin. RBP4; retinol binding protein-4. Mg; milligrams. L; litre. G; grams. dL; decilitre. Mmol; millimoles. µg; microgram. µmol; micromole*
